# A bibliometric analysis of diabetic gastroparesis from 1979 to 2024

**DOI:** 10.3389/fmed.2024.1445276

**Published:** 2024-10-10

**Authors:** Hui Xu, Furui Miao, Yushan Fan

**Affiliations:** College of Acupuncture-Moxibustion and Tuina, Guangxi University of Chinese Medicine, Nanning, China

**Keywords:** diabetic gastroparesis, diabetes mellitus, bibliometrics, CiteSpace, VOSviewers

## Abstract

**Objective:**

Gastroparesis is one of the complications of diabetes mellitus, which has a major impact on the quality of life of patients, and the limited therapeutic options currently available make it a public health problem. No bibliometric studies on diabetic gastroparesis have been published to date. Therefore, the aim of this paper is to summarize and analyze the research hotspots for researchers.

**Methods:**

Research articles related to Diabetic gastroparesis were searched in Web of Science Core Collection (WOSCC), and relevant information was extracted after screening. A comprehensive bibliometric analysis of 699 publications was conducted using Microsoft Excel 2019, Citespace and VOSviewers.

**Result:**

A total of 699 papers from 738 institutions in 41 countries were retrieved. Publications in this field have increased rapidly since 1979. USA (*n* = 370) and Mayo Clinical (*n* = 69) were the most productive country and institution, respectively. Neurogastroenterology and Motility (*n* = 67) was the most published journal with Parkman, Henry P. (*n* = 40) having the highest number of articles; Gastroenterology and Mccallum, Richard W. were the most influential journals and authors.

**Conclusions:**

The research hotspots of Diabetic gastroparesis are mainly focused on treatment modalities and pathological mechanisms. Future research in diabetic gastroparesis will focus on exploring the pathomechanisms, finding long-term effective treatments, and improving patients' quality of life.

## 1 Introduction

Diabetic gastroparesis (DGP) is one of the common clinical complications of diabetes mellitus, which is mainly manifested by nausea, vomiting, abdominal distension, and other symptoms characterized by decreased gastrointestinal motility and delayed gastric emptying (1). Approximately 9.3% of individuals diagnosed with diabetes experience gastroparesis (2). Gastroparesis can have negative implications for the management of diabetes mellitus in both adults and adolescents (3). This condition can result in gastrointestinal malabsorption issues, electrolyte imbalances, malnutrition, and other complications. It can also heighten the risk of cardiovascular disease, significantly impacting the quality of life for patients (4). Currently, the primary treatment for diabetes mellitus involves medications and surgical interventions, which can lead to drowsiness, cardiac side effects, and adverse extrapyramidal diseases (5). Several researchers have investigated the therapeutic approaches for DGP and have discovered that acupuncture (6), ginseng pectins (7), mulberry (8), and oolong tea (9) have shown efficacy in treating the condition. Hence, it is imperative to investigate the current areas of research and emerging patterns in the domain of DGP in order to offer fresh guidance for the management and treatment of DGP.

In recent years (10–13), researchers have extensively utilized bibliometrics to study various diseases such as cancers, osteoarthritis, myasthenia gravis, and COVID-19. It becomes an invaluable tool for researchers to track hotspots, trends and guide clinical practice. Notably, the WoSCC database contains more than 12,000 of the most influential journals, making it ideal for bibliometric analyses. This study employed bibliometric techniques to examine publications on DGP from 1979 to 2024, with the objective of identifying prominent areas of research and trends in the field. Additionally, the study aimed to offer novel insights and proposals for future research on DGP.

## 2 Methods

### 2.1 Data sources and search strategies

We retrieved articles on diabetic gastroparesis from the Web of Science Core Collection (WOSCC) and the Social Sciences Citation Index (SSCI). The search covered the time from the inception of the databases to May 12, 2024, based on the database build date. We imposed constraints on the document type, allowing just articles, confined the language to English, and excluded publications that had been retracted. The detailed search formula is as follows: TS=(‘Diabet^*^ Gastropares^*^') AND DT=(Article) AND LA=(English).

### 2.2 Data extraction and analysis

A total of 699 documents were retrieved and exported from the WoSCC database as plain text files in the format of “complete records and citations” for analysis using bibliometric tools. Graphs were drawn using Microsoft Excel 2019 to predict the growth trend of publications using the available data. Visual maps of collaborative networks and keyword clusters were constructed using VOSviewer to analyse general publication characteristics including year of publication, author, country, region, organization and citation frequency. Use CiteSpace to identify research hotspots and cutting-edge trends in a time dimension. The “time sliding” value was set to 1 year, and the node type was selected according to the purpose of the analysis. In this study, we used “country, institution, journal, author, citation frequency, keyword” as the node type for visual analysis, and only one node type could be selected at a time.

## 3 Results

The study included 699 publications from 41 countries, involving 738 institutions, 3,044 authors, and 257 journals. Among these, 16 publications were mentioned more than 50 times. A total of 13,375 papers from 2,946 journals were cited, and 2,306 keywords were observed. Encompasses the fields of gastroenterology, hepatology, pharmacology, pharmaceuticals, neurology, endocrinology, and metabolism.

### 3.1 Publication analysis

**Figure 1:**
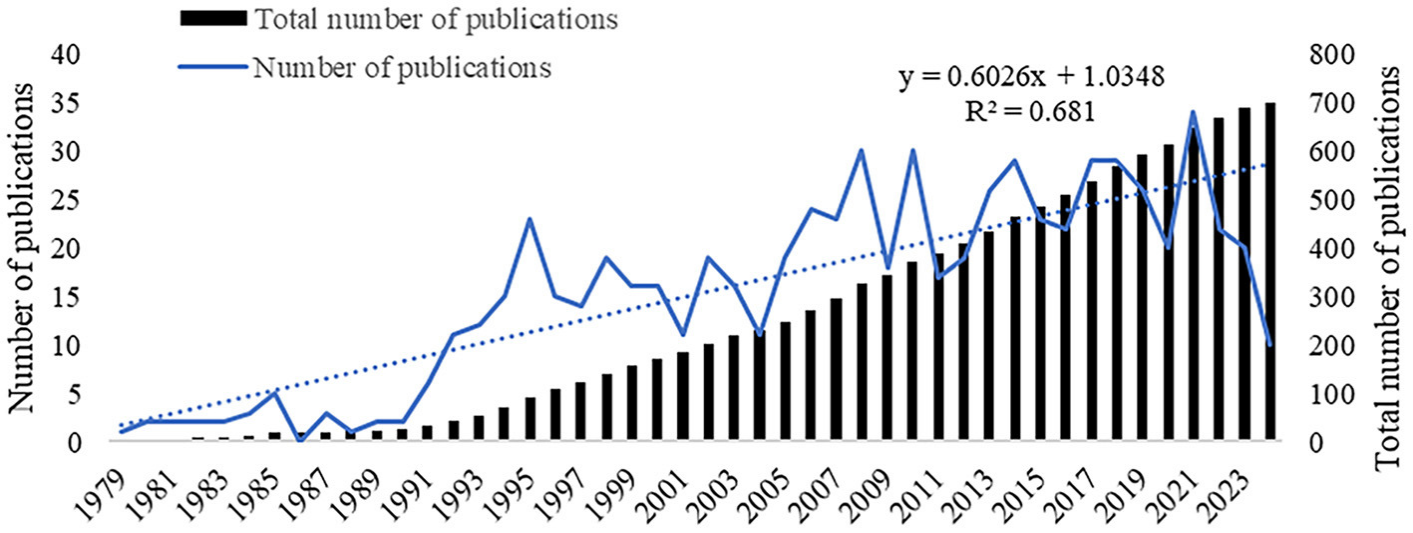
Trends in DGP article publication.

In this study, a comprehensive search was conducted on a total of 699 papers. Figure 1 illustrates the annual and cumulative count of relevant publications that were published. Since the publication of first article on DGP in 1979, the annual number of papers on this topic has remained low until 2005. Research on DGP is still in its nascent phase. Between 2005 and 2021, there was a variable and rapid growth in the number of papers published, reaching its highest point in 2021 with 34 publications. The figure below shows the overall stability of the number of publications on articles pertaining to diabetic gastroparesis.

### 3.2 Country analysis

**Figure 2:**
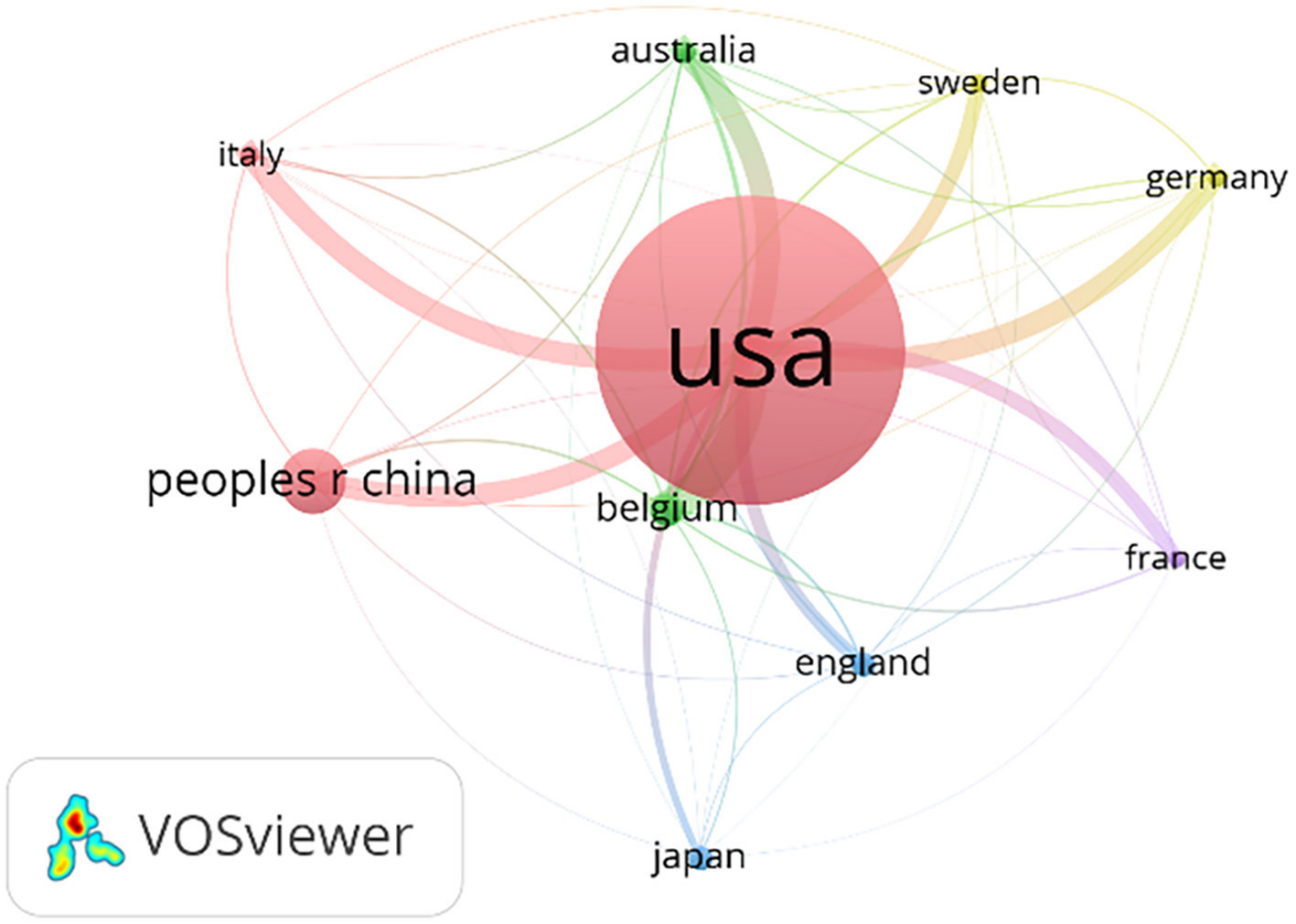
Network diagram of partnerships for national launches.

A total of 41 countries were involved in publications related to DGP in 699 publications, and the Table 1 lists the top 10 countries that published articles, with the USA, China, and Belgium ranking in the top three, with the USA having a total of 339 publications, accounting for about half (48.5%) of the publications, and with an average citation frequency of about 49.7. The number of China publications is 88, accounting for about 12.6% and ranking second, with an average citation frequency of about 10.20. Australia ranked sixth among them with 15 publications, yet boasts a high average citation frequency of 61.00, indicating the high quality and reference value of the articles. Regarding the analysis of the cooperation network, the Figure 2 shows that the USA is the country with the widest range of cooperation. The USA cooperates most closely with Australia, Italy, Germany and Belgium.

**Table 1:** Ranking of DGP-related articles by country of publication.

**Country**	**Publications**	**Total citations**	**Average citation per publication**
USA	370	18,715	50.58
China	80	664	8.30
Belgium	40	3,832	95.80
England	29	1,286	44.34
Japan	29	747	25.76
Australia	26	2,339	89.96
Italy	24	1,774	73.92
Germany	22	1,750	79.55
France	20	1,499	74.95
Sweden	19	1,325	69.74

### 3.3 Contributions of institutions

**Figure 3:**
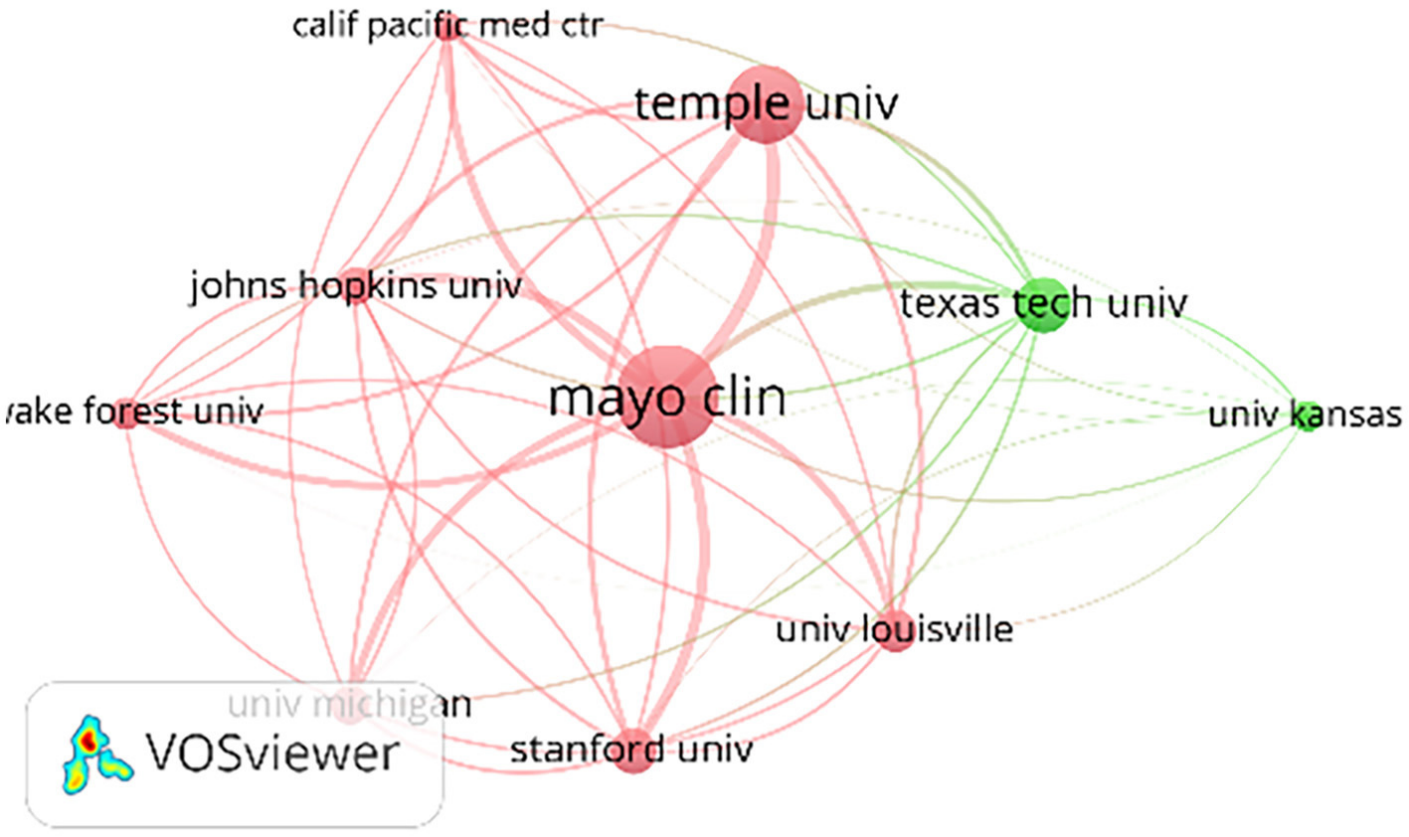
Map of institutional cooperation networks for the issuance of documents.

A total of 738 organizations are engaged in the publication of articles about DGP. Table 2 presents the ranking of the top 10 institutions in the field of DGP, based on the quantity of articles published. Mayo Clinical, Temple University, and Texas Tech University occupy the top three positions in this ranking. It is worth mentioning that all of the top 10 institutions are associated with schools and institutes in the USA. Figure 3 displays a map illustrating the collaboration network among the top 10 organizations. It reveals that Mayo Clinical has the broadest scope of collaborations and maintains the closest partnerships with Temple University, Stanford University, and Johns Hopkins University.

**Table 2:** Top 10 research organizations in terms of publications in the DGP field.

**Institutions**	**Publications**	**Total citations**	**Average citation per publication**
Mayo Clinical	69	3,729	54.0435
Temple University	53	2,200	41.5094
Texas Tech University	37	979	26.4595
Stanford University	31	1,625	52.4194
University of Louisville	29	922	31.7931
University of Michigan	27	1,605	59.4444
Johns Hopkins University	26	1,154	44.3846
Wake Forest University	22	945	42.9545
University of Kansas	21	2,185	104.0476
California Pacific Medical Center	20	1,001	50.05

### 3.4 Contributions of journals

A total of 699 articles were published in 257 journals, and Table 3 lists the top 10 journals in terms of the number of articles published, as well as their citation frequency and Impact Factor (IF). The journal with the highest number of publications was Neurogastroenterology and Motility, with 67 articles. Digestive Diseases and Sciences followed with 52 articles. Gastroenterology had the highest total citation frequency (*n* = 3,804) and average citation frequency (*n* = 115.27), with an impact factor (IF) of 29.4. The American Journal of Gastroenterology, with an IF of 9.8, had a total citation frequency of 2,460 and an average citation frequency of 91.11.

**Table 3:** Top 10 journals in terms of number of published articles.

**Journals**	**Publication**	**Total citations**	**Average citation per publication**	**IF (2023)**
Neurogastroenterology and Motility	67	1,682	25.10	3.5
Digestive Diseases and Sciences	52	2,324	44.69	3.1
Gastroenterology	33	3,804	115.27	29.4
Alimentary Pharmacology & Therapeutics	28	1,675	59.82	7.6
American Journal of Gastroenterology	27	2,460	91.11	9.8
American Journal of Physiology-Gastrointestinal and Liver Physiology	16	851	53.19	4.5
Gastrointestinal Endoscopy	11	525	47.73	7.7
Gastroenterology Clinics of North America	10	334	33.40	3.7
Journal of Diabetes and its Complications	10	176	17.60	3
Clinical Gastroenterology and Hepatology	9	584	64.89	12.6
Journal of Clinical Gastroenterology	9	462	51.33	2.9

### 3.5 Contributions of authors

**Figure 4:**
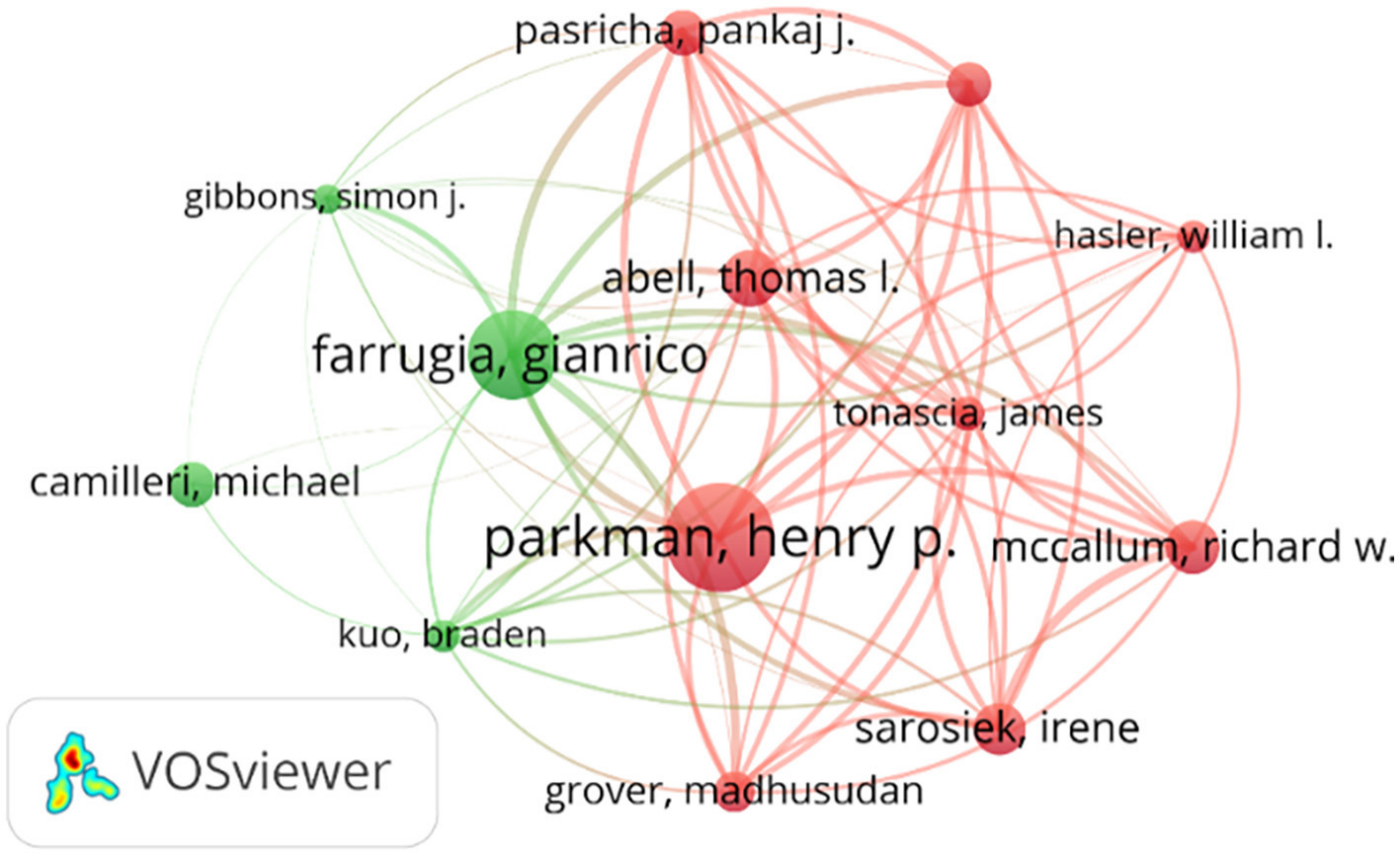
Network diagram of the top 10 cited authors' collaborations.

The number of publications in the DGP field by an author represents the extent of his contribution to the field. The total number of citations reflects the influence of the authors. Table 4 presents the top 10 authors in terms of publications in the DGP field, as well as the total number of citations for their publications and the average number of citations per paper. Evidently, Parkman, Henry P. has the most number of publications with 40, a total citation of 1,692, and an average citation per publication of 42.30. Followed by Farrugia, Gianrico, Abell, and Thomas L. Farrugia, Gianrico has 33 total publications with a total citation of 2,230 and an average citation per publication of 67.58. Abell, Thomas L. has 21 publications with a total citation of 725 and an average citation per publication of 34.52; McCallum, Richard W. has the highest average citation per publication of 106.61; Using the collaborative network diagram in Figure 4, we find that Sarosiek, Irene, has the strongest collaborative links with McCallum, Richard W.

**Table 4:** Top 10 authors in terms of publications in the DGP field.

**Authors**	**Publications**	**Total citations**	**Average citation per publication**
Parkman, Henry P.	40	1,692	42.30
Farrugia, Gianrico	33	2,230	67.58
Abell, Thomas L.	21	725	34.52
Mccallum, Richard W.	20	534	26.70
Sarosiek, Irene	19	339	17.84
Mccallum, R. W.	18	1,919	106.61
Camilleri, Michael	17	824	48.47
Frrugia, Gianrico	17	1,098	64.59
Koch, Kenneth L.	16	668	41.75
Grover, Madhusudan	15	672	44.8

### 3.6 Citations

The citation frequency of a paper can reflect the influence of the paper in the field; the higher the frequency, the greater the influence. Table 5 lists the 10 most cited articles in the field of DGP, about half of which discuss the treatment of DGP. Janssens J. in 1990 published “Improvement of gastric emptying in diabetic gastroparesis by erythromycin. Preliminary studies” suggested that erythromycin has a significant mitigating effect on delayed gastric emptying in patients with DGP, possibly by acting as a gastric agonist through binding to the gastric actin receptor. The study of erythromycin on DGP was initiated. Yeo CJ published “Erythromycin accelerates gastric emptying after pancreaticoduodenectomy. A prospective, randomized, placebo-controlled trial” in Annals of Surgery in 1993, verified the effect of erythromycin on delayed gastric emptying. Thomas Abell published “Gastric electrical stimulation for medically refractory gastroparesis” in Gastroenterology in 2003, noting the ameliorative effect of gastric electrical stimulation on diabetic gastroparesis. The mechanism by which ghrelin improves DGP was explored in “Influence of ghrelin on interdigestive gastrointestinal motility in humans,” published by Tack J. in GUT in 2006. Simultaneously, researchers were conducting studies on the pathophysiological mechanisms of DGP. In 1984, Camillerim M. identified concomitant abnormalities of intestinal peristalsis in DGP patients, expanding their focus beyond the stomach. Grover M. published “Cellular changes in diabetic and idiopathic gastroparesis” in Gastroenterology in 2011, in which a cytological study of diabetic gastroparesis was carried out, and found that the majority of patients with gastroparesis were found to have cellular abnormalities, Kit expression deficiencies, ICC deficiencies, and increased immunoreactivity for CD45 and CD68. In 2009, Gomez-Pinilla P. J. published “Ano1 is a selective marker of interstitial cells of Cajal in the human and mouse gastrointestinal tract” in the American Journal of Physiology—gastrointestinal and liver physiology. “Ano1 is a selective marker of interstitial cells of Cajal in the human and mouse gastrointestinal tract” published in the American Journal of Physiology-gastrointestinal and Liver Physiology in 2009, pointed out that in addition to Kit as a marker of ICC, Ano1 can also be a marker of ICC. Regarding the diagnosis of DGP, Revicki DA, in 2003, published “Development and validation of a patient-assessed gastroparesis symptom severity measure: the Gastroparesis Cardinal Symptom Index,” in which the article proposes a new method for the assessment of gastroparesis by creating the GCSI scale to rate the severity of symptoms of gastroparesis.

**Table 5:** The top 10 documents in citation analysis of publications in DGP.

**Title**	**Journals**	**Years**	**First author**	**Total citations**	**TC per year**	**Normalized TC**
Improvement of gastric emptying in diabetic gastroparesis by erythromycin. Preliminary studies	New England Journal of Medicine	1990	Janssens J	624	17.83	1.99
Gastric electrical stimulation for medically refractory gastroparesis	Gastroenterology	2003	Abell, Thomas L.	391	17.77	5.27
Erythromycin accelerates gastric emptying after pancreaticoduodenectomy. A prospective, randomized, placebo-controlled trial	Annals of Surgery	1993	Yeo C. J.	335	10.47	4.15
Gastric pacing improves emptying and symptoms in patients with gastroparesis	Gastroenterology	1998	Mccallum, Richard W	324	12.00	6.32
Gastric emptying and gastric myoelectrical activity in patients with diabetic gastroparesis: effect of long-term domperidone treatment	American Journal of Gastroenterology	1989	Koch, Kenneth L.	299	8.31	1.84
Ano1 is a selective marker of interstitial cells of Cajal in the human and mouse gastrointestinal tract	American Journal of Physiology-gastrointestinal And Liver Physiology	2009	Gomez-Pinilla P. J.	293	18.31	7.51
Abnormal intestinal motility in diabetics with the gastroparesis syndrome	European Journal of Clinical Investigation	1984	Camillerim M.	287	7.00	2.22
Cellular changes in diabetic and idiopathic gastroparesis	Gastroenterology	2011	Grover M.	282	20.14	5.13
Influence of ghrelin on interdigestive gastrointestinal motility in humans	GUT	2006	Tack J.	277	14.58	5.15
Development and validation of a patient-assessed gastroparesis symptom severity measure: the Gastroparesis Cardinal Symptom Index	Alimentary Pharmacology & Therapeutics	2003	Revicki D. A.	267	12.14	3.60

### 3.7 Keywords analysis

**Figure 5:**
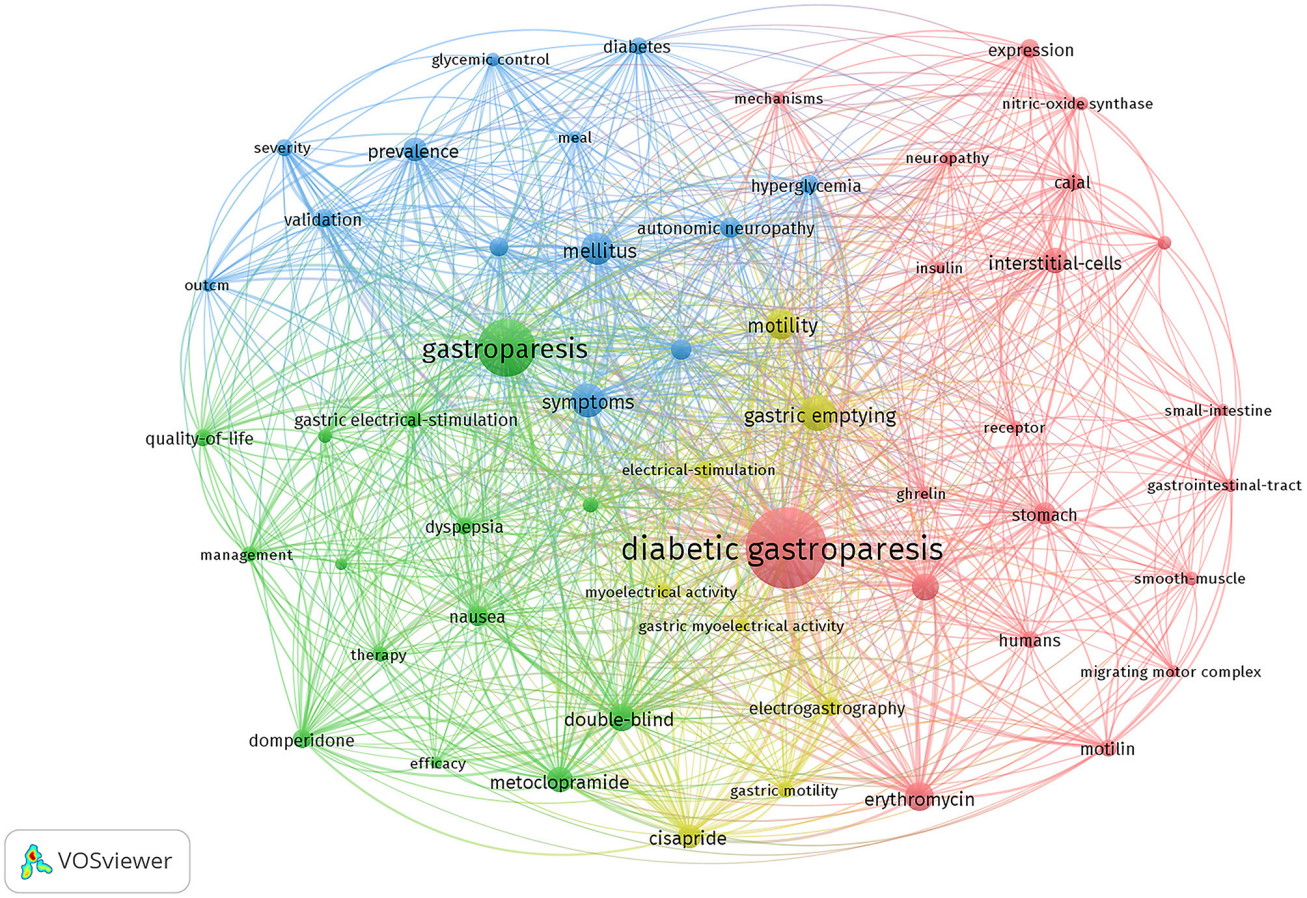
Co-occurrence network visualization map of keyword.

**Figure 6:**
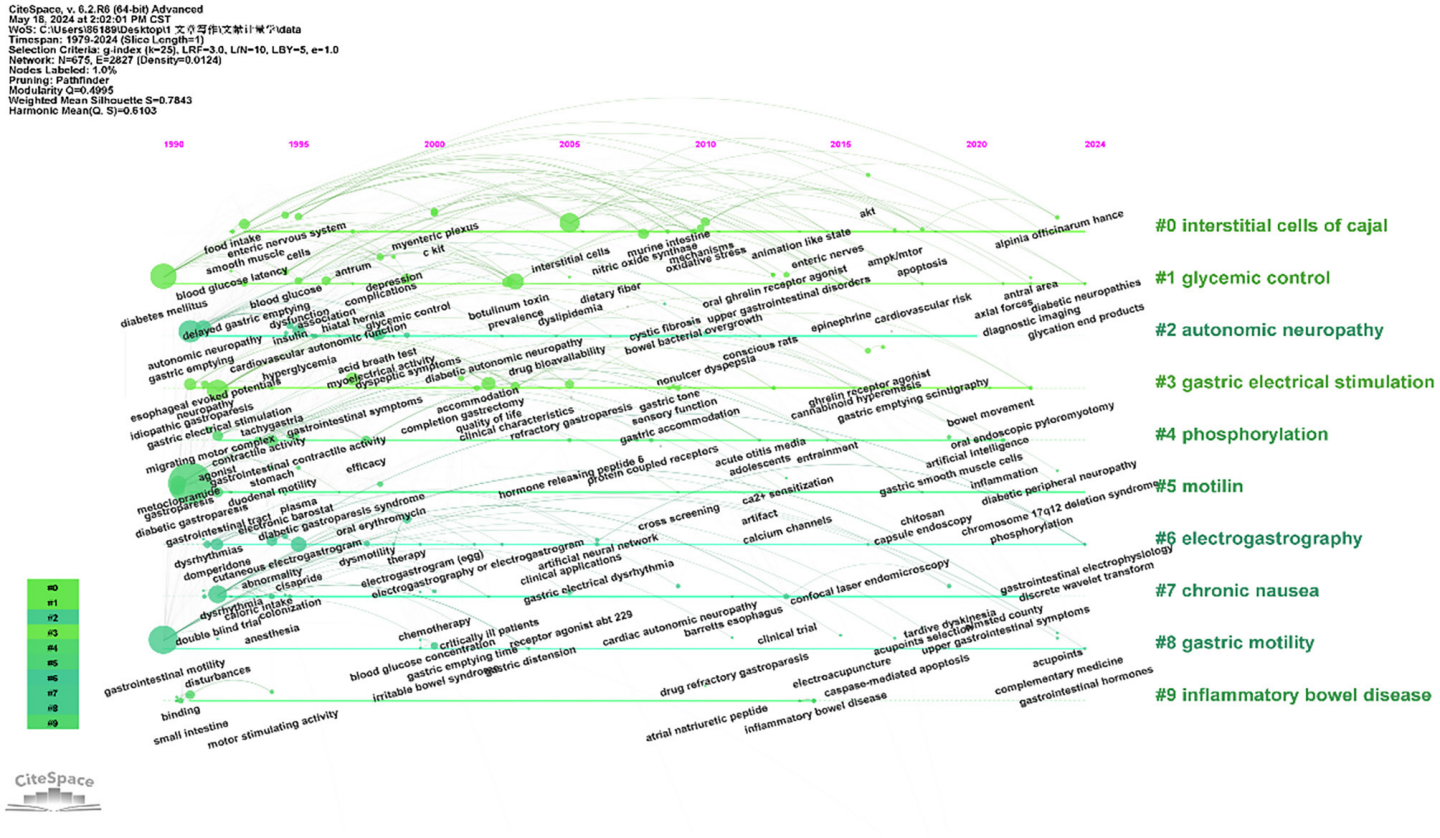
Timeline view of reference keywords analysis.

In indexing or cataloging, a keyword is a concise word that accurately and succinctly describes the topic of an article. Table 6 shows the top 20 keywords in terms of frequency of occurrence in the DGP domain. The 57 keywords that appeared more than 20 times are shown in Figure 5, except for common words such as “diabetic gastroparesis”, “gastroparesis”, and “gastric emptying.” The four main research directions of DGP are indicated by four colors: green for therapeutic clustering, red for pathological mechanism clustering, blue for epidemiological clustering, and yellow for diagnostic clustering. Figure 6 depicts the distribution of keywords over time. By tracking the transitions of each keyword, one can visualize the migratory route of the research focus. The clusters are categorized as “interstitial cells of cajal,” “glycemic control,” “autonomic neuropathy,” “gastric electrical stimulation,” “phosphorylation,” and so on.

**Table 6:** Top 20 keywords with frequency of occurrence in DGP field.

**Keywords**	**Occurrence**	**Keywords**	**Occurrence**
Diabetic gastroparesis	382	Functional dyspepsia	60
Gastroparesis	229	Metoclopramide	60
Gastric emptying	104	Prevalence	57
Symptoms	96	Cisapride	56
Mellitus	94	Stomach	51
Motility	85	Diabetes mellitus	47
Erythromycin	78	Autonomic neuropathy	45
Gastrointestinal motility	71	Nausea	44
Double-blind	68	Hyperglycemia	42
Interstitial-cells	66	Domperidone	40

**Figure 7:**
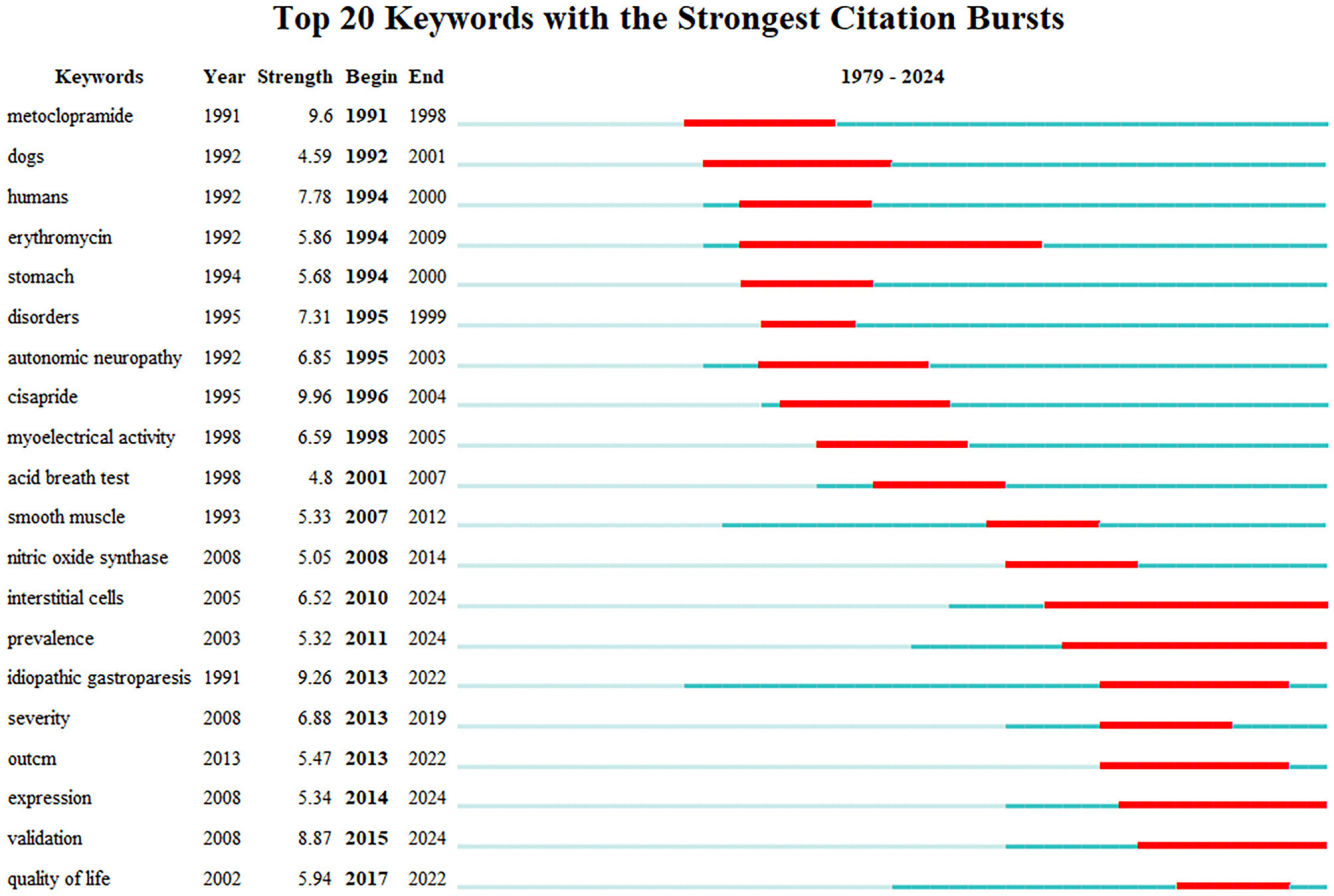
Top 20 keywords with the strongest citation bursts in DGP.

The term “keyword burst” refers to keywords that are frequently cited over a period of time. Thus, it indicates the frontier area. Figure 7 shows the 20 bursts of keywords sorted by “start year.” As shown, the heat of investigation related to the keywords “erythromycin,” “intestitial cell,” and “prevalence” has continued for more than 10 years. The keywords cisapride (9.96) and metoclopramide (9.6) are in the top rankings. The keywords that have continued to the present day are “intestitial cell,” “prevalence,” “expression,” and “validation.”

## 4 Discussion

### 4.1 Trends in the prevalence of DGP

Diabetes and its complications are now a major global health problem. Currently, 1 in 10 people have diabetes, and by 2030, it is predicted that around 643 million people, or 11.3% of the global population, would have diabetes. This number is projected to rise to 783 million, or 12.2% of the population, by 2045 (14, 15). In the last 15 years, there has been a 316% rise in worldwide health expenditure on diabetes. Gastroparesis is a prevalent consequence of long-term high blood sugar levels in individuals with diabetes, affecting around 9.3% of diabetic patients (2). At the same time, gastroparesis can cause changes in the rate of food emptying, leading to fluctuations in blood glucose, again exacerbating the symptoms of gastroparesis, with blood glucose and gastroparesis interacting with each other (16). The disease severely impairs patients' quality of life (17) and increases hospitalization rates (18–20).

We conducted a systematic and comprehensive bibliometric analysis of studies on DGP from 1979 to the present using Vosviwer and CiteSpace software. The first document retrieved was published in 1979, which addressed the effectiveness of oral metoclopramide in the treatment of diabetic gastroparesis. This was followed by a series of articles on DGP. In 1984, Camillerim M. et al. conducted an analysis of the extensively referenced literature and observed that the impaired movement of the digestive system known as dysmotility of DGP affects not only the stomach but also involves the duodenum in the underlying pathological processes. In 1994, Yeo C. J. et al. discovered that erythromycin has the ability to expedite gastric emptying. As a result, they initiated research on the potential application of erythromycin in the treatment of diabetic gastroparesis. As the diabetic patient population grew due to population growth and economic development, there was a corresponding increase in the number of patients with diabetic gastroparesis. Researchers started studying the causes of this condition around 2005, and by 2021, the number of scientific papers on diabetic gastroparesis had reached its highest point. The United States leads in the number of publications, representing around 50% of relevant articles. Additionally, the United States has high research quality and is the most prominent country in the field of DGP research. Simultaneously, the USA puts significant emphasis on scientific collaboration and has forged cooperative partnerships with multiple countries in DGP research. This may also contribute to the exceptional research quality of its publications. Historically, the majority of DGP research undertaken before the 20th century was mostly focused in industrialized nations such as the United States, Australia, and Germany. With the rise in economic growth and the growing aging population in China, there has been an increase in the number of diabetic patients. Consequently, research on DGP has been steadily carried out in developing countries. In approximately 2014, China initiated research on DGP, and currently, a significant proportion of published papers are from China. However, there is room for improvement in the quality of these studies. All of the top 10 institutions in the field of DGP are either universities or medical research institutions located in the United States. Among these, the Mayo Clinic stands out for having the biggest number of published articles and the most extensive network of collaborations with other institutions. The University of Kansas has fewer than one-third of the amount of articles compared to Mayo Clinic, although its average citation rate is around double that of Mayo Clinic's, indicating its research is highly valuable.

Henry P. Parkman is the most published author and is affiliated with Temple University; Richard W. McCallum is ranked second and is affiliated with Johns Hopkins University; and Farrugia Gianrico is ranked third and is affiliated with Mayo Clinic College of Medicine. The top 10 authors published a total of 198 DGP-related articles, accounting for ~28.3% of the total number of DGP publications. McGallum, Richard W. is the author with the highest average number of citations and has made outstanding contributions to the field of DGP, conducting research in the field of DGP in 1983 and proposing the important role of gastric pacing in facilitating gastric emptying and improving the symptoms of patients with gastroparesis in 1998. In 1998, he suggested that gastric pacing has an important role in promoting gastric emptying and improving symptoms in patients with gastroparesis. The most published journal is Neurogastroenterology and Motility with 67 articles, followed by Digestive Diseases and Sciences with 52 articles. Among the top 10 journals publishing DGP-related papers, six journals had a JCR partition of Q1, which on the one hand indicates the importance of diabetic gastroparesis and on the other hand shows the high quality of research in DGP despite the low number of DGP papers. Geoenterology has the highest impact factor and average citation frequency, indicating that articles published in this journal are credible and well-referenced. The above study can be used as a reference for researchers in the field of DGP when selecting journals.

### 4.2 Keywords

DGP (21) refers to the gastrointestinal symptoms associated with diabetes mellitus. It is also known as gastroparesis, a syndrome characterized by decreased movement in the gastrointestinal tract and a delay in the emptying of the stomach. Diabetic gastroparesis is characterized by more pronounced symptoms (22), reduced quality of life, and an elevated mortality risk (23) compared to other forms of gastroparesis. Regarding cytoarchitectural abnormalities, DGP patients commonly exhibit smooth muscle cell alterations and thickening of the basal lamina around nerves, whereas patients with idiopathic gastroparesis more frequently show perineural fibrosis (24).

Gastric emptying scintigraphy (GES) is widely regarded as the most reliable diagnostic technique for identifying DGP (25). In 2003, researchers utilized the 13C-octanoic acid breath test to measure the pace at which the stomach empties in diabetes patients. This test has a specificity of 0.73 and is currently commonly employed for diagnosing delayed gastric emptying due to its safety and simplicity (26). Upper gastrointestinal endoscopy is the first step in ruling out other potential diagnoses, but if food remains are found in the stomach after an overnight fast, this is suggestive of diabetic gastroparesis. Other diagnostic modalities include stable isotope gastric emptying breath test (GEBT), capsule endoscopy, electrogastrography (EGG), and functional ultrasound (5).

#### 4.2.1 Pathological mechanisms of DGP

The timeline graph indicates that the clusters of intestitial cell of cajal (ICC), autonomic neurpathy, phosphorylation, and Motilin are the keywords or terms that appear most frequently in the literature. This reflects the physiopathological mechanisms of DGP as a prominent and actively researched area. According to the timeline diagram, ICC began to appear in the 1990s, when researchers discovered the role of gastric pacing in promoting gastric emptying in patients with gastroparesis and that ICC generates electrical pacemaker activity and mediates motor neurotransmission in the stomach (27–30). C-kit, as a receptor tyrosine kinase, is a marker of ICC expression, and stem cell factor SCF is a ligand for kit, between which signaling is necessary for the development and maintenance of ICC and is essential for gastric electrical pacing activity in the (28, 31, 32). In the 21st century, a large number of studies have been conducted on the mechanisms of ICC in diabetic gastroparesis. Visible ICC depletion in diabetic patients and its consequences by causing smooth muscle atrophy and reduced SCF production (33). At the same time, the reduction of ICC also causes a decrease in neuronal nitric oxide synthase (nNOS) and substance P-containing (SP) nerve expression (34). Exogenous SCF treatment increases SCF levels, c-kit expression, and ICC number in diabetic rats (35).

The nerves that control the movement of the digestive tract are mostly autonomic nerves. These nerves are made up of two main systems, sympathetic and parasympathetic, which can control the functions of internal organs directly or indirectly. The parasympathetic nervous system has both excitatory and inhibitory control over gastrointestinal motility, so a malfunction of this control can lead to disturbed motility patterns, resulting in nausea, vomiting, and even delayed gastric emptying (36). Patients with DGP have autonomic dysfunction, and autonomic neuropathy scores are positively correlated with the gastric emptying rate of solid food. Reduced nerve density and abnormal morphology of the gastric mucosa in DGP patients were found by gastric mucosal biopsy (37). Gou C. et al. found that the number of cells in the motor vagal ganglia and sensory sympathetic ganglia of the gastrointestinal tract of diabetic rats was reduced by controlled experiments in a rat model of streptozotocin-induced diabetes with age-matched healthy controls.

Oxidative stress (38) refers to an imbalance between the production of reactive oxygen species (ROS) and reactive nitrogen species (RNS) and the removal of antioxidant defenses in the body, resulting in the excessive production of ROS and RNS, which causes damage to tissues, cells, and macromolecules such as proteins and nucleic acids. damage to tissues, cells, and biomolecules such as proteins and nucleic acids. Heme oxygenase-1 (HO-1) is an important cytoprotective molecule against oxidative damage. Loss of HO-1 upregulation in diabetic mice presenting with delayed gastric emptying. CD206(+) M2 macrophages exhibit the expression of HO-1. The presence of a higher number of macrophages and the elevation of HO-1 in CD206(+) M2 macrophages are closely linked to the onset of diabetes mellitus. Additionally, a specific reduction in CD206(+)/HO1(+) M2 macrophages can be detected in cases of delayed stomach emptying (39). With the in-depth study of the mechanism, oxidative stress has become a hot spot in the study of diabetic gastroparesis. Increased ROS content in gastric tissues of rats with diabetic gastroparesis (40). Factor E2-associated factor 2 (Nrf2) is involved in the induction of antioxidant enzymes, and Nrf2 is closely related to immune regulation and oxidative stress. Hyperglycemia induces apoptosis by elevating pro-inflammatory cytokines, ROS, and inhibiting neuronal nitric oxide synthase via PI3K/Nrf2-mediated signaling (41). Delayed gastric emptying in diabetic patients is also associated with gastrointestinal hormonal disorders, neuroimmunity, and intestinal flora (42), and the exploration of the pathophysiological mechanisms of DGP remains a hot topic of research.

#### 4.2.2 Treatment of DGP

##### 4.2.2.1 Glycemic control

There is an interaction between glycemic control and delayed stomach emptying in diabetic patients (43). Hyperglycemia delays gastric emptying of solid and liquid foods compared to normoglycemia (44), especially in a state of chronic hyperglycemia (45). Hyperglycemia causes osmotic stress, inflammatory changes, and damage to the small blood vessels supplying the nerves, further contributing to neuropathy in diabetic patients (46). Interventions that slow gastric emptying (e.g., glucagon-like peptide-1 receptor agonist) reduce postprandial blood glucose (47). Glycated hemoglobin reflects the patient's level of glycemic control over the last 3 months. Jorge Calles- Glycemic management was demonstrated to be advantageous for patients with DGP by Calles-Escandon et al. (48), who used continuous subcutaneous insulin infusion and continuous glucose monitoring to observe glycated hemoglobin, gastroparesis symptoms, and quality of life in these patients. For diabetic patients with gastroparesis, treatment with insulin induces hypoglycemia and a higher risk of hypoglycemia compared to patients without gastroparesis. Recent studies have found that the development of a hybrid closed-loop system reduces the burden of diabetes management in patients with T1D, enables glucose-responsive insulin delivery, and protects against hypoglycemia, resulting in improved glycemic control (49–51).

##### 4.2.2.2 Improvement in gastric motility

According to the frequency of keywords, excluding the repeated words such as diabetic gastroparesis, gastroparesis, mellitus, etc., the words with the highest frequency of occurrence are gastric emptying, symptoms, motility, erythromycin, gastrointestinal motility, and other words. Also, gastrointestinal motility had the highest centrality, followed by gastric emptying. In 1990, Jansses J. confirmed that erythromycin provides significant relief of delayed gastric emptying in patients with diabetic gastroparesis. Molitin (42) causes contraction of the gastric body and sinus a and is able to promote gastric emptying, whereas erythromycin binds to gastric motility receptors and acts as an agonist. Chini et al. (52) found that azithromycin was more capable of inducing anterior activity of the gastric sinus and thus duodenal contractions, as well as a longer duration of action as compared to erythromycin. In the 1970s, researchers found that metoclopramide improved gastric emptying in patients with DGP (53). Metoclopramide, as a dopamine receptor antagonist, improves gastric emptying by enhancing peristalsis in the duodenum and jejunum, and at the same time has a centrally produced antiemetic effect. The U.S. Food and Drug Administration (FDA) has approved it as the sole medication for treating gastroparesis. However, its use is currently limited due to its high number of adverse effects (54). Domperidone is also a dopamine receptor antagonist and has a very low risk of extrapyramidal adverse effects compared to metoclopramide because it does not cross the blood-brain barrier (55). Gastric hunger hormone agonists are novel prokinetic drugs for the treatment of gastric bradycardia, and Relamorelin belongs to the group of gastric hunger hormone agonists that improve nausea, abdominal pain, postprandial fullness, and bloating in patients with DGP, and accelerate the rate of gastric emptying (56). In the 1990s, cisapride was widely used to improve gastric motility in patients with DGP (57–59). However, it is rarely used today because of its side effects on the heart. Gastric electrical stimulation, used since the 1970s, improves gastric symptoms through continuous electrical stimulation of the gastric sinuses, especially in patients for whom other therapies have failed (60). STZ-induced impaired gastric motility and reduction of gastric electrical slow waves in diabetic rats, and GES was found to normalize gastric motility in diabetic rats (61). Other treatment modalities include endoscopic modalities such as intra-pyloric injection of botulinum toxin, transoral gastroendoscopic myotomy, and surgical modalities such as pyloroplasty, gastrectomy, etc. (21).

##### 4.2.2.3 Treatment goal

Quality of life has been shown to be a strong predictor of survival. Studies have found nausea, and as the number of people with diabetes increases, the quality of life of people with diabetes has been an issue that researchers have been working on (62). Living with diabetes often carries a significant psychosocial cost, which can negatively impact self-care behaviors, long-term glycemic control, and the risk of long-term complications. Symptoms of vomiting and abdominal pain as well as comorbidities such as anxiety and depression and psychological factors all contribute to a reduced quality of life for DGP patients (63, 64). Researchers in clinical care are increasingly concerned about patients' quality of life.

### 4.3 Limitations

This paper examines the literature on diabetic gastroparesis through bibliometric and visualization analyses, and there are some limitations. First, only English literature was included in this study, which may lead to biased findings. Second, we only retrieved data from the WoSCC database and did not retrieve information from other databases (e.g., PubMed, Scopus, etc.), which resulted in a potentially incomplete collection of literature. Third, we did not include all keywords related to DGP when conducting the literature search. In order to gain a more comprehensive understanding of the field, future studies could build on this to refine the search strategy, resulting in stronger evidence support. Fourth, there is no systematic standard in setting up the parameter settings and analysis methods of the CiteSpace software, which may lead to discrepancies in the results.

## 5 Conclusion

This study represents the initial and comprehensive bibliometric analysis of diabetic gastroparesis. It examines the focal points and cutting-edge areas of research on diabetic gastroparesis, including publications, countries, institutions, journals, authors, and keywords. Research on diabetic gastroparesis has gained significant attention from researchers in the last 40 years. Understanding the mechanisms of diabetic gastroparesis, exploring treatment options, and enhancing the quality of life for patients are ongoing and important topics of study. The study's findings provide insights into potential areas of future research focus and assist researchers in identifying suitable research collaborators and academic publications.

## Data Availability

The original contributions presented in the study are included in the article/supplementary material, further inquiries can be directed to the corresponding author.
